# ‘It's especially good just to know that you're not the only one’: a qualitative study exploring experiences with online peer support programmes for the Fragile X community

**DOI:** 10.1111/jir.13188

**Published:** 2024-09-25

**Authors:** T. Haber, L. Davies, R. S. Hinman, K. L. Bennell, W. Bruce, L. Jewell, A. Borda, B. J. Lawford

**Affiliations:** ^1^ Centre for Health, Exercise and Sports Medicine, Department of Physiotherapy, School of Health Sciences University of Melbourne. Victoria Melbourne Victoria Australia; ^2^ Fragile X Association of Australia Sydney New South Wales Australia; ^3^ Centre for Health Policy Melbourne School of Population and Global Health Melbourne Victoria Australia

**Keywords:** disability, fragile X, fragile X syndrome, online, peer support, qualitative

## Abstract

**Background:**

Accessing peer support can be difficult for people with, or carers of people with, inherited intellectual disabilities. One way to improve access is to provide services online, yet few studies have explored people's experiences with online peer support programmes. We aimed to explore experiences with such programmes for communities affected by fragile X‐associated conditions.

**Methods:**

Qualitative study involving individual semi‐structured interviews with 16 people with, or carers of people with, a fragile X‐associated condition (*n* = 4 adult premutation carriers; *n* = 12 parents/carers of children/adults), who participated in at least one of three online peer support programmes: educational webinars, Facebook discussion group and small peer group sessions via Zoom. Reflexive thematic analysis was used to develop themes.

**Results:**

Three overarching themes relating to experiences were as follows: (1) uncertainty and value of shared experiences, (2) support navigating healthcare, (3) advantages being online, but still a place for in‐person events. Educational webinars were perceived to be a valuable source of information about fragile X‐associated conditions although people had variable information needs. Facebook discussion groups enabled people to connect with others, although participants expressed some competing preferences for how the groups were organised. Zoom peer group sessions were perceived to help participants feel supported by others, but that consistency in organisation was important.

**Conclusions:**

Online peer support programmes were perceived to be beneficial, bridging informational gaps and facilitating social connection. However, participants believed there was still a place for in‐person events, some felt educational webinars did not always meet their needs and some had privacy concerns.

## Introduction

Inherited intellectual disorders can cause physical, intellectual, and social disability, impacting those affected in various ways, including causing emotional difficulties, behavioural symptoms, and developmental delays (Scallan *et al*. 2011; Kayadjanian *et al*. 2018; von der Lippe *et al*. 2022). The most common inherited cause of intellectual disability (ID) is fragile X syndrome, caused by a change to a gene on the X chromosome (Hunter *et al*. 2014; Better Health Channel 2022) which causes a wide range of physical, intellectual and behavioural symptoms and impacts an estimated 1 in 5000 people in Australia (Hunter *et al*. 2014). Other genetic conditions that can cause ID include Rett syndrome, Angelman syndrome, Prader–Willi syndrome and Williams syndrome.

People with inherited genetic disorders may share common health problems, but often, even within conditions, have unique health needs (von der Lippe *et al*. 2022). For example, while disorders typical to fragile X syndrome include low muscle tone, autism spectrum disorder, anxiety and attention‐deficit hyperactivity disorder (Ciaccio *et al*. 2017; Better Health Channel 2022), individuals often experience these with varied degrees of severity ranging from mild to severe. Individuals with fewer gene changes may be diagnosed as a fragile X premutation carrier, with some experiencing health problems such as early menopause, adult‐onset neurodegenerative disorders (e.g. ataxia), and mental health and social problems (e.g. anxiety and shyness) (Gallagher & Hallahan 2012).

Information about inherited genetic conditions is often complex, requiring people to understand genetics and various health issues arising from the condition (Centers for Disease Control and Prevention 2022). Unfortunately, accessing specialised multidisciplinary healthcare providers with knowledge of these relatively rare genetic conditions can be challenging (Scallan *et al*. 2011; Chaij *et al*. 2014; von der Lippe *et al*. 2022). Indeed, those who are affected often feel poorly understood by health professionals and uninformed about managing the condition and what to expect in the future (Gane *et al*. 2010; Scallan *et al*. 2011; von der Lippe *et al*. 2022). People with, or caring for those with, genetic conditions thus want more information to increase their confidence in managing health issues now and in the future (Centers for Disease Control and Prevention 2022; von der Lippe *et al*. 2022).

People affected by inherited genetic conditions value peer support networks and contact with other families affected by the same or similar conditions (Gane *et al*. 2010; von der Lippe *et al*. 2022). Given that families of people with these conditions, such as fragile X, can face a lifetime of caretaking challenges (Bailey *et al*. 2008; Bailey *et al*. 2012), peer support between families is a vital way of sharing resources and ideas and providing emotional support (Gane *et al*. 2010; Centers for Disease Control and Prevention 2022). One of the biggest challenges identified by parents of children with fragile X syndrome is the lack of knowledge about the condition in the community, making it difficult to form support networks that can respond to their needs and to explain the condition to other family and friends (Minnes & Steiner 2009). Furthermore, people with inherited genetic conditions, and their carers, often find it challenging to access support networks and engage in social activities due to barriers such as a lack of time or carer responsibilities (Gane *et al*. 2010; von der Lippe *et al*. 2022).

One way to improve access to information and peer support networks for people living with, or supporting an individual affected by, inherited intellectual disorders is to deliver them online (Raspa *et al*. 2018). The vast majority of research examining online peer support groups for people with disabilities have focused on Autism spectrum disorders (Sehlin *et al*. 2018; Abel *et al*. 2019; Oudshoorn *et al*. 2020; Sartore *et al*. 2021), and to our knowledge, none have specifically focused on those with inherited intellectual disorders, including the most common of these, fragile X syndrome. Given the unique educational and emotional support needs of people with these conditions (e.g. complex genetic information, lack of community knowledge, lifetime of caretaking challenges, lack of time and carer responsibilities), further research into the role of online peer support groups for these communities is necessary.

Fragile X Association of Australia is the peak body for people living with fragile X‐associated conditions and their families and/or carers. The organisation is a registered charity and supported by a small number of staff, eight volunteer directors and its members (see https://www.fragilex.org.au/about‐us/). Since COVID‐19, the Fragile X Association of Australia has introduced several online peer support group programmes for its members and the wider Australian fragile X community: (1) group peer support sessions via Zoom (designed to enable sharing of information and experiences between small groups of people, with discussions facilitated by a qualified counsellor); (2) an online educational webinar series (delivered by invited speakers to provide education about fragile X syndrome, fragile X premutation and their associated health conditions); and (3) a Facebook peer discussion group (sharing information from both the association and peer‐to‐peer, including upcoming events and resources relevant to the community). This study aimed to explore the experiences of people with, or carers of someone living with, a fragile X‐associated condition who have engaged in these online peer support programmes.

## Methods

### Design

A qualitative design based on an interpretivist paradigm was undertaken. According to this paradigm, knowledge about a phenomenon is developed by gathering the perceptions and interpretations of participants who experience or are affected by it (Thanh & Thanh 2015). A phenomenological framework was used (Groenewald 2004), focusing on the lived experiences of people involved with the issue being researched. The Consolidated Criteria for Reporting Qualitative Research checklist was used to ensure complete and transparent reporting (Tong *et al*. 2007). The University of Melbourne Institutional Human Research Ethics Committee approved this study, and participants gave informed consent.

### Participants

People who were fragile X premutation carriers, or are a parent/carer of someone with a fragile X‐associated condition, were recruited. Between May and August 2022, the Fragile X Association of Australia ran advertisements for the study on their social media, website, emails and via word of mouth within their community. Participants were eligible if they were >18 years of age and had participated in at least one of three peer support programmes run by the Fragile X Association of Australia (i.e. Facebook group, webinars or group Zoom sessions) in the last 6 months. Potentially interested participants were invited to complete a short online eligibility survey. If eligible, they were contacted by a member of the research team and an interview day/time was booked.

Once approximately 50% of interviews were complete, the demographics of the sample were reviewed, and subsequent invitations were targeted to ensure diversity concerning online peer support experiences and participant residence in metropolitan/regional areas of Australia. We ceased recruiting when we deemed we had reached theoretical saturation– where no new themes emerged from the data (Bowen 2008).

### Fragile X Association of Australia peer support programmes

This study focuses on experiences with three programmes (described Table 1): (1) group peer support sessions via Zoom; (2) an online educational webinar series; and (3) a Facebook peer discussion group. Briefly, Zoom group peer support sessions were designed to enable sharing of information and experiences. Groups included up to six attendees, with discussions being facilitated by a qualified counsellor. Online educational webinars aimed to provide education about fragile X syndrome, fragile X premutation and their associated health conditions. Webinars were held approximately once per month and were delivered by invited speakers (e.g. clinicians and researchers). The Facebook peer discussion group involves peer‐to‐peer sharing of information and resources relevant to the community, moderated by staff or volunteers from the Association. The group has ~1,000 members.

**Table 1:** Description of the online peer support programmes provided by the Fragile X Association of Australia

Zoom group peer support sessions	Online education webinars	Facebook peer discussion group
**Delivery:** Sessions are facilitated by a staff member (a qualified counsellor) and involve up to 6 participants. Session can run for up to 1 h in duration. **Purpose:** Members can use peer support groups to share information and experiences and provide emotional support to one another. The fragile X Association of Australia organises group members, intending to match participants purposefully (e.g. people with some similar experiences of fragile X – such as children of the same age/gender or individuals who have fragile X‐associated ataxia syndrome) or match new carers of children recently diagnosed with fragile X with more experienced carers who can share the experiences, perspectives, and insights. **Frequency:** Zoom support groups are organised ad hoc by staff members, who will contact members that may be appropriate for those groups as they are being organised. Some groups met regularly (e.g. once a month ongoing), while other groups comprise different members each time they are run.	**Delivery:** Presented by invited speakers (e.g. expert clinicians and researchers in the field) and facilitated by staff of the Association. Webinars enable some interaction by allowing attendees to ask questions. Webinars can last up to 1 h. **Purpose:** To learn more about fragile X syndrome, fragile X premutation and their associated health conditions. Webinar topics have included ‘Understanding Behaviours and Fragile X Syndrome’, ‘Sensory Processing and Fragile X’ and ‘Daily Living Skills and Learning Strategies’. The webinars also allow some interaction between members, expert researchers and clinicians in the field. Webinars can be tailored to various members, including people with fragile X disorders and their families, carers and healthcare providers. **Frequency:** Approximately once a month.	**Delivery:** Private Facebook peer discussion group for those affected by fragile X disorders. The group has close to 1,000 members, 75% of whom are Australians. **Purpose:** The Facebook group predominantly involves peer‐to‐peer sharing of information and resources about fragile X disorders or issues relevant to the fragile X community – for example, discussions around the education system, National Disability Insurance Scheme, social supports, referrals and events. The Facebook group is a primary way that the Association shares information and upcoming events (e.g. webinars) with its members. The Facebook group also allows members to connect to form social and support networks. **Frequency:** The Facebook page is moderated daily by staff or volunteers from the Association.

### Interviews

We used a semi‐structured interview guide (Table 2) to explore participants' experiences with key elements of the online peer support programmes. Interviews were conducted via Zoom by TH, a physiotherapist and researcher trained in qualitative methodologies with no connection to participants or the Fragile X Association of Australia, nor any lived experience of fragile X‐associated conditions. Interviews lasted 30–45 minutes and were audio recorded and transcribed verbatim by an external provider of transcription services. All data were de‐identified and stored in digital format on a password‐protected university server.

**Table 2:** Semi‐structured interview guide

Preamble: We are interested in finding out about your experiences with the online peer support programmes that are run by the Fragile X Association of Australia and your thoughts on those experiences. There are no right or wrong answers. We are very keen to hear your opinions or reactions, positive or negative or neutral. Please stop me at any time if you need to take a break, or want to end the interview for any reason. Also, you don't have to answer every question – just let me know if you want to move on from something. Before I start, do I have your permission to audio‐record this session? ***Zoom peer support groups*:** Have you participated in any of the Zoom peer support groups organised by the Fragile X Association of Australia? *[if NO to Q1] Why not? Is there anything that might make you more inclined to participate?* [if YES to Q1] how did you find that experience? What did you like about it? What didn't you like about it? How has it made any difference to your health/wellbeing? [if parent/carer] how has it made any difference to the person with the fragile X disorder? Will you continue to participate in the future? Why/why not? What could be improved for future use? ***Educational webinars*:** Have you participated in any of the educational webinars organised by the Fragile X Association of Australia? *[if NO to Q1] Why not? Is there anything that might make you more inclined to participate?* [if YES to Q1] how did you find that experience? What did you like about it? What didn't you like about it? How has it made any difference to your health/wellbeing? [if parent/carer] how has it made any difference to the person with the fragile X disorder? How did you feel about the different topics covered by the webinars? Are there any other topics you think should be covered? Will you continue to participate in the future? Why/why not? What could be improved for future use? ***Facebook online support group*:** 1 Have you participated in the Facebook online support group organised by the Fragile X Association of Australia? *[if NO to Q1] Why not? Is there anything that might make you more inclined to participate?* 2 [if YES to Q1] how did you find that experience? 3 What did you like about it? 4 What didn't you like about it? 5 How has it made any difference to your health/wellbeing? [if parent/carer] how has it made any difference to the person with the fragile X disorder? 6 Will you continue to participate in the future? Why/why not? What could be improved for future use? *Final question/s*: 7 Are there any other online health supports that you think the Fragile X Association of Australia, or other healthcare providers, should offer to help support the fragile X community?

Questions in italics were used as prompts if necessary.

### Data analysis

We undertook inductive reflexive thematic analysis (Braun & Clarke 2019) using NVivo version 12 software (Jackson & Bazeley 2019). Transcripts were first read multiple times, with and without audio recordings, before being coded separately by T. H. and L. D. This consisted of line‐by‐line data analysis and identifying elements that appeared important to the research question. Both researchers then independently refined the codes by adapting, merging and sorting them into a structure representing themes that related to overall participant experiences with online peer support programmes, as well as their experiences with each individual programme. T. H. and L. D. then met to discuss emerging categories and ideas. Themes and sub‐themes were reviewed multiple times to ensure external heterogeneity and internal homogeneity. To ensure the credibility and confirmability of the data, another researcher (B. J. L.) read all transcripts before meeting with T. H. and L. D. to discuss and review the themes that had been developed.

## Results

Interviews were undertaken with four fragile X premutation carriers (age range 21–50 years) and 12 parents/carers of children or adults with a fragile X‐associated condition (Table 3). Eight (50%) participants were female, 10 (63%) were residents of metropolitan areas, 8 (50%) worked full‐time and most (88%) had tertiary education.

**Table 3:** Characteristics of interview participants (*n* = 16)

	Type of participant	Online peer support programmes participated in	Age of person affected	Gender of person affected	Geographic location	Employment status^†^	Highest level of education^†^	Frequency of social media use^†^	Frequency of videoconferencing use^†^
P1	Parent/carer	Webinars	41‐50 years	Male	Rural/regional	Retired	Postgraduate degree	Every day	Once a week
P2	Premutation carrier	Webinars Facebook group	21–30 years	Female	Rural/regional	Not employed	Some high school	Every day	Once a week
P3	Premutation carrier	Webinars	31–40 years	Female	Rural/regional	Other:	Some high school	Every day	Once a month
P4	Parent/carer	Webinars	21–30 years	Male	Rural/regional	Retired	Postgraduate degree	Every day	Once a month
P5	Premutation carrier	Group Zoom Webinars Facebook group	41–50 years	Female	Metropolitan	Not employed	Postgraduate degree	Every day	Several times a week
P6	Parent/carer	Group Zoom Webinars	0–10 years	Male	Metropolitan	Work full‐time	Bachelor's degree	Every day	Every day
P7	Parent/carer	Group Zoom Facebook group	21–30 years	Male	Rural/regional	Work part‐time	Bachelor's degree	Every day	Once every few months
P8	Parent/carer	Group Zoom Facebook group	11–20 years	Male	Metropolitan	Work full‐time	Bachelor's degree	Every day	Every day
P9	Parent/carer	Group Zoom Webinars	11–20 years	Female	Metropolitan	Work part‐time	Postgraduate degree	Once a week	Every day
P10	Parent/carer	Group Zoom	61–70 years	Male	Rural/regional	Retired	Postgraduate degree	Every day	Once a week
P11	Parent/carer	Webinars	0–10 years	Male	Metropolitan	Other:	Bachelor's degree	Every day	Several times a week
P12	Parent/carer	Webinars	0–10 years	Female	Metropolitan	Work full‐time	Postgraduate degree	Once every few months	Several times a week
P13	Parent/carer	Group Zoom	0–10 years	Female	Metropolitan	Work full‐time	Bachelor's degree	Every day	Every day
P14	Premutation carrier	Webinars Facebook group	41–50 years	Female	Metropolitan	Other:	Bachelor's degree	Every day	Once a week
P15	Parent/carer	Webinars	0–10 years	Female	Metropolitan	Work part‐time	Bachelor's degree	Every day	Once every few months
P16	Parent/carer	Group Zoom Webinars Facebook group	11–20 years	Male	Metropolitan	Work part‐time	Bachelor's degree	Every day	Several times a week

^†^ Of the interviewee.

### Overarching themes

Three overarching themes relating to all online support programmes were developed and are outlined below:

#### Uncertainty and value of shared experiences

Participants used the peer support programmes to address uncertainty about what lies ahead – for themselves and for those they are caring for. The peer support programmes helped manage this uncertainty by drawing on the experiences of others affected by fragile X (e.g. parents learning from other parents with children older than their own). People also valued the ability to share these experiences, which they saw as both benefiting others and as a key coping strategy for themselves. In connecting with others with similar lived experiences, participants of these programmes no longer felt ‘alone’.

P2: For doctors and stuff, it's easy to say, ‘This is how it is,’ but then when you live it it's different … I didn't know anything, and it was just amazing to be able to feel connected, and I got a lot of valuable information and resources and stuff.

P13: It was really good, so I think I called [name removed] the day after my first daughter was diagnosed and she was very kind and talked to me on the phone and then she organised for me to meet up with, on Zoom, I think another family who has a girl who is a few years older, just to connect and ask questions about what does this mean and what is it like.

#### Support navigating healthcare

Many participants struggled to find information about fragile X after they, or someone they cared for, was diagnosed. Often, they were dissatisfied with a lack of education about fragile X from healthcare professionals, perceiving a lack of knowledge or expertise about the condition in the healthcare system. The peer support programme bridged the gap in these informational needs in two ways: (1) by providing information about fragile X‐associated conditions and how to manage them (both self‐manage and manage within the healthcare system) and (2) by connecting people with healthcare professionals who have expertise in managing fragile X‐associated conditions.

P1: People are like, ‘What's that? What's Fragile X?’ and I have to tell them, it's a genetic thing – if it's the leading cause of autism, how do people not know this?

P12: Being premutation there is not a lot that can be done for us, and we get fobbed off, and we're not acknowledged by the NDIA we're not acknowledged by doctors … I have to manage it all myself, I have to come up with my own solutions, I'm just dealing with everything by myself.

P16: I always try and communicate it with the whole support team with the boys, different things. And it gave me the opportunity to connect to my son's speech therapist with the speech therapy, the session therapy. I don't know, because she wasn't really using strategies that were engaging for my son, like the high interest things and stuff like that.

#### Advantages being online, but still a place for in‐person events

All participants valued the online delivery of peer support programmes, particularly their convenience and accessibility. Accessing programmes from home often suited participants with busy schedules – many carers reported a lack of time as a barrier to many activities. Being online also allowed those from across all regions of Australia to attend and connect with others they would not otherwise meet. Furthermore, some felt that participating online was particularly beneficial for helping them cope with anxiety as they felt it was okay to limit their contributions (e.g. turn their camera off) or leave the session if they felt overwhelmed. However, despite these benefits, participants desired some in‐person support programmes – feeling that in‐person events could better foster personal connections and make it easier to interpret important non‐verbal cues.

P4: The other good thing about the webinars is you know, we used to have a lot of in person meetings, but they were particularly Sydney or Melbourne focused. And so that, you know, COVID forcing us to go down the webinar format has actually widened the audience that can access that information.

P7: I also really value about the webinars the question so you do not actually have to – you can just put your question in a chat, so you do not feel like, I do not know, everyone is looking at you.

P13: There's something really valuable about being with people in person that it's kind irreplaceable. So I'd like that, and it'd be really nice actually if you could just have more of them as well.

##### Themes related to the positives and drawbacks of each type of support programme

**Figure 1:**
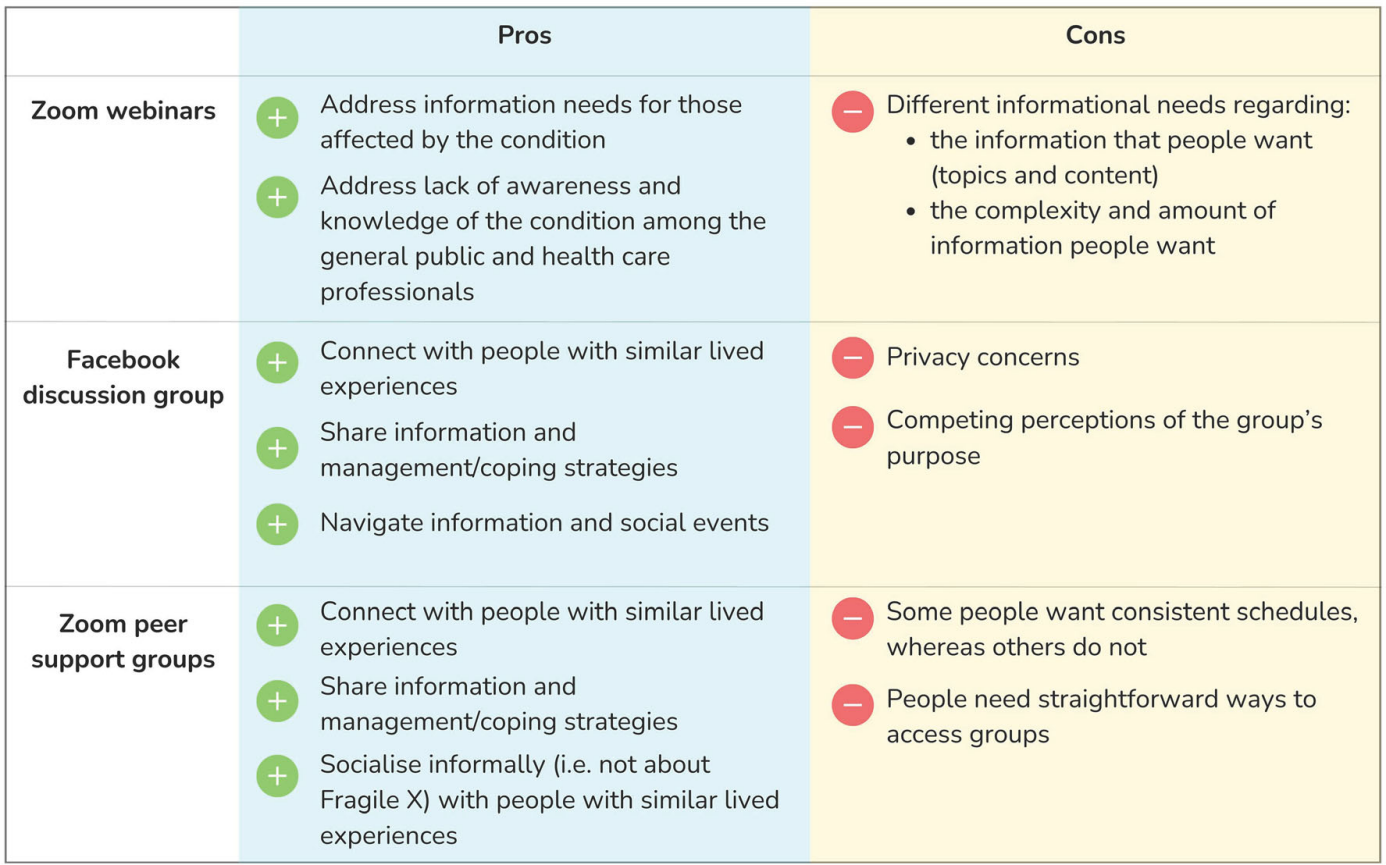
Positives and drawbacks of different online peer support programmes.

Two themes distinct to each type of support programme were developed. Figure 1 highlights the positives and drawbacks of each peer support programme. Notably, participants valued the Facebook discussion group and Zoom group peer support sessions for sharing information/coping strategies and socialising with people with similar lived experiences. Participants felt that educational webinars were distinctly important for addressing knowledge gaps among the general public and healthcare professionals. Drawbacks were often specific to each programme type. Key concerns voiced by participants included that Zoom webinars could not always address different information needs, such as information relevant to people with fragile X of differing ages. For the Facebook page, some were concerned about privacy and competing perceptions of the programme's purpose or organisation. Lastly, for the Zoom peer support group sessions, some wanted easier ways of accessing these groups. Furthermore, participants had conflicting preferences, with some wanting consistent scheduling for the Zoom group while others did not. These positives and drawbacks are described in more detail for each programme support type below.

#### Experiences with education webinars

##### Valuable source of information about, and management strategies for, fragile X‐associated conditions

Participants perceived a general lack of knowledge about fragile X‐associated conditions within the community, making them feel isolated and unsupported by those around them. Participants felt that these webinars were an essential resource addressing these informational gaps, particularly valuing information about understanding and managing behaviours (relating to fragile X‐associated syndrome), for example, supporting informed decision making on medication use, and strategies for self‐advocating within the healthcare system.

P10: The [webinar] topics have been, like, really bang on. The experts that they've brought in have been really, really good. So – and so informative. So yeah, fantastic experience.

P10: Three of our support workers attended the webinars too, which was awesome. They raved about them. They felt –they really raved about it helping to understand what Fragile X is and how it presents.

#### Variable informational needs

Informational needs of participants varied, and thus, some felt that there were topics that were inadequately addressed in the webinars. For example, a carer of an adult male with a fragile X‐associated condition felt that most information was aimed at children or female premutation carers, making some of the content not directly relevant. Further, some participants also found the amount or complexity of information in some webinars made it challenging to comprehend and absorb.

P12: As premutation, no, I don't feel very heard, and I don't feel very understood – I do really find that there has just been – such a lack of focus and interest in the girls, that's really hard, and being a mum of a Fragile X is really hard because I don't feel I have a lot of support or help around that.

P1: Most of it up ‘til now, as far as I could see was aimed at either children or female carriers, and so not adult males. And probably because we're the first ones of the adult males to be adults, sort of thing.

P5: Sometimes practically it's hard because your particular issue or their particular issue may [differ], if that makes sense to you, and you're sort of flying a bit blind as to help each other.

#### Experiences with Facebook peer discussion group

##### Connect with others and events

People valued the Facebook page as a way to connect with other families affected by fragile X, helping grow their support networks. Some also used the Facebook page to share experiences and learn from others, such as providing or receiving advice about navigating healthcare and understanding behaviours. The Facebook page was often the primary way people navigated Fragile X Association of Australia services and other fragile X‐related information or social events.

P4: I think my experience, because my son's a bit older, you know, some people have benefited from my experience – now they are asking really the same questions I did as my own son was growing up … it's not only answers to your question, but it's also feeling like you're not alone, that other people have gone through it.

P2: Even like the GP and everything like that sort of printed off some info, but they didn't really know about it – they weren't, so to sort of go on there, it was like I could talk to people that have lived it, been through it, or just understood it.

P3: Well, I mean, that's basically – even though I'm on the email list for all these webinars and things that come out, being on the Facebook page makes it a lot easier. They send – they provide the links and reminders and stuff like that, so that's been really helpful as well in making sure I'm not missing anything that I want to be involved with.

#### Competing preferences

Some participants felt that the Facebook group was too formal as there was a lack of sharing of personal stories or experiences. In contrast, other participants were wary of sharing information on the Facebook page, citing privacy concerns. These conflicting views highlighted a tension in how participants viewed the Facebook group.

P5: One thing that would probably be of benefit – some groups have a ‘ask anonymously’ type question sort of thing. Because I do feel that privacy issue is a big one at the moment with Facebook.

P12: In those [other] forums, people will actually get on there and ask really personal questions – And so it really gets into that community nitty gritty, and we don't really have that kind of forum in the Fragile X Association anywhere.

#### Experiences of Zoom group peer support sessions

##### Being supported by and supporting others

Participants valued Zoom peer support groups as a medium to share their experiences with others and provide, or receive, advice on how to manage certain aspects of living with or caring for someone with a fragile X‐associated condition. Some used the Zoom groups as general catch‐up – a way of informally socialising with people with similar lived experiences.

P6: The support group was helpful for my mental health. To know that there are other mums out there that have the same struggles that most people don't understand, or you just don't even bother trying to get them to understand because they have no idea.

P7: Just feeling like somebody actually understands and even just those ‘me too’ moments where other parents would be like ‘oh my kid does this’ or whatever and you are like ‘oh yes’, all the time. Or just feeling like other parents understand and there is that solidarity and you're not as alone, I think. Because no one – even in your friendship circles – once people know you have the diagnosis, they do not know anything about it and they usually [don't] ask.

##### Consistency and organisation are important

Participants had suggestions regarding how online peer support programmes are organised and advertised to the community. For example, some felt unsure of how to sign up for Zoom groups. Others believed Zoom groups could be held more frequently or regularly, viewing consistency as necessary due to their busy schedules.

P11: I do sometimes get the Zoom invites quite last minute, so – you know, with work schedules and things, obviously, the more notice, the better.

P12: We need to just have them more regularly, maybe I haven't been paying attention and there have been more and I haven't seen them.

P10: It might be nice to have a once‐a‐month mums catch‐up or something, rather than a one‐off, because I think it's nice if things are organised – and we're reminded to catch up.

## Discussion

We found that participants valued all online peer support programmes as a source of information about fragile X‐associated conditions and how to manage them; many felt that these programmes addressed their informational needs unmet by healthcare professionals. Notably, in addition to being educational tools, the peer support programmes were seen as a beneficial way to connect with others and share experiences. However, each of the three programmes – educational webinars, Facebook peer discussion group and Zoom peer support sessions – had specific positives and drawbacks (Figure 1).

A key finding was that the information provided via the online peer support programmes was convenient and accessible. Consistent with our findings, those with, or caring for those with, fragile X and other genetic conditions, such as Rett syndrome (McGraw et al. 2023), Prader–Willi syndrome (Chaij et al. 2014) and Williams syndrome (Scallan et al. 2011), often find it difficult to access social and professional support due to a lack of knowledge among the public, teachers, support workers (Bailey et al. 2008; Van Remmerden et al. 2020). Further, they often view healthcare providers as lacking expertise in managing these conditions (Visootsak *et al*. 2016; Van Remmerden *et al*. 2020). Findings from this study suggest that one way to enhance the accessibility of information about these conditions is to promote expert knowledge online. Similarly, other research have found that those living with or caring for people with disabilities believe that online peer support programmes gave them opportunities to participate and connect, which were not otherwise possible because they lacked access to in‐person activities (Banbury *et al*. 2019; Barclay & Lalor 2022). However, similar to our findings, participants in those studies also believed that there was still a place for in‐person peer support (Banbury *et al*. 2019; Barclay & Lalor 2022). Interestingly, in contrast to prior work (Banbury *et al*. 2019; Barclay & Lalor 2022), our participants did not perceive any technological barriers to participation in online peer support programmes. A potential reason for this difference is that participants in this study reported a relatively high level of technology literacy (Table 3). Future research should explore the perceptions of people with fragile X who report lower levels of technology literacy to determine if these individuals encounter challenges accessing online peer support programmes.

We found that participants felt well supported by the online support programmes and could establish strong connections with others, suggesting that the mode of service delivery did not inhibit social interaction. Indeed, consistent with prior research (Banbury *et al*. 2019), we found that many participants felt that engaging in activities online helped reduce their social anxiety, allowing them some relative anonymity (e.g. ability to turn the camera off if uncomfortable). This is important given that it is common for people with inherited genetic conditions, or their carers, to experience mental health issues (Barnes *et al*. 2015; Kayadjanian *et al*. 2018; Keary *et al*. 2022), such as those living with fragile X‐associated conditions who can experience social anxiety, shyness and social avoidance (Hagerman *et al*. 1992; Bennetto *et al*. 2001).

One of the key benefits of the online peer support programmes was the sense of connectedness to others with similar experiences, many of whom they may otherwise have never met. Indeed, our findings, and past research in those with fragile X and other rare genetic and neurodevelopmental conditions, show that connecting with others helps alleviate distress arising from uncertainty about the future, particularly among carers of young children (Lewis *et al*. 2006; McCarthy *et al*. 2006; Hartley *et al*. 2012; Van Remmerden *et al*. 2020; von der Lippe *et al*. 2022), and from the pressures of ‘constant’ caring responsibilities (Van Remmerden *et al*. 2020). Systematic reviews of peer support programmes for carers of children with disabilities found evidence that such programmes can reduce distress, improve wellbeing and quality of life and increase feelings of emotional and informational support, particularly when coupled with education (Wallace *et al*. 2021; Lancaster *et al*. 2022).

Our findings also demonstrate that via online delivery, a small organisation like the fragile X Association of Australia – a registered charity with only two employees – can deliver education and support to a national community comprising people with varying family, carer and social responsibilities and health problems. Similar organisations representing people affected with inherited genetic disorders could use our findings to inform the design and delivery of their own peer support programmes – for example, by using a range of formats (i.e. social media, group meetings via videoconferencing and informational webinars), guided by the potential strengths and weaknesses of each format (Figure 1). Notably, people affected by, or caring for those, with genetic or neurodevelopment conditions, such as fragile X‐associated conditions, often have varied information needs (Redley *et al*. 2018). For example, we found that some participants felt they needed more information targeted at adult men with fragile X syndrome while others thought they needed more information relevant to female Fragile X premutation carriers. Our findings thus suggest that organisations and service providers should consider the specific needs of individuals within their communities, which might differ based on how they are impacted by a given health problem, what types of information they want or need (e.g. level of detail) and, if they are caregivers, the ages and needs of those they care for. Future research should evaluate whether experiences with online peer support programmes differ between those affected by a fragile X‐associated condition and those that are a carer or parent of someone affected.

Our study also highlights opportunities to improve future online peer support programmes. Consistent with research in online support groups for people with adults and youth with disabilities (Banbury *et al*. 2019; Cassiani *et al*. 2020; Barclay & Lalor 2022), many participants were keen to maintain some in‐person activities, suggesting a hybrid delivery of support groups may be beneficial. Participants also believed it was important to ensure that peer support programmes are clearly and consistently organised, such as making the sign‐up for activities or the finding of relevant information clear. Organisations supporting communities affected by disability could explore preferences and needs for online peer support programme within their community, including people's desire to maintain in‐person activities and their preferences for level of organisation for support services. However, as connectivity issues, low technology confidence or lack of compatible devices can deter or prevent people from using online services (Banbury *et al*. 2019; Barclay & Lalor 2022), addressing these barriers may be another important area for future research.

### Study limitations

Our study has strengths and limitations. Strengths include the broad sample of people interviewed, including adult males and females from metropolitan and regional areas, and carers of adults and children of varying ages with a fragile X‐associated condition and fragile X premutation carriers. Limitations of our study include that our sample comprised participants who volunteered to participate in this qualitative study. Thus, we may have captured people with mostly positive views of the programme. In addition, our participants were all based in Australia, spoke English and were primarily tertiary educated (i.e. 88% had tertiary education). Therefore, our findings may not be transferable to those with lower levels of education, non‐English speaking countries or linguistically diverse communities within Australia or other high‐income countries. Finally, we did not use strategies like member checks or data triangulation, which may have impacted the credibility and dependability of our findings.

## Conclusion

Online peer support programmes for communities affected by this inherited genetic disorder were perceived to be beneficial, bridging informational gaps, providing practical information regarding management and healthcare and facilitating social connection among people with similar lived experiences. However, participants believed there was still a place for in‐person events, some felt educational webinars did not always meet their needs, and some had privacy concerns with online programmes. Other organisations could use these findings to guide the development and implementation of their own peer support programmes.

## Conflict of interest

None to declare.

## Source of Funding

This study is funded by a Melbourne Disability Institute grant. R. S. H. is supported by a National Health & Medical Research Council Fellowship (#1154217) and KLB by a NHMRC Investigator Fellowship (#1174431).

## Ethics statement

The University of Melbourne Institutional Human Research Ethics Committee approved this study (#24166).

## Data Availability

Data are available upon reasonable request to the corresponding author.
